# Cytotoxicity and Genotoxicity Assessment of Ascorbyl Palmitate (AP) Food Additive

**DOI:** 10.15171/apb.2018.039

**Published:** 2018-06-19

**Authors:** Yousef Sohrabi, Hossein Mohammadzadeh-Aghdash, Elham Baghbani, Parvin Dehghan, Jafar Ezzati Nazhad Dolatabadi

**Affiliations:** ^1^Immunology Research Center, Tabriz University of Medical Sciences, Tabriz, Iran.; ^2^Department of Food Science and Technology, Faculty of Nutrition and Food Sciences, Nutrition Research Center, Tabriz University of Medical Sciences, Tabriz, Iran.; ^3^Student Research Committee, Tabriz University of Medical Sciences, Tabriz, Iran.; ^4^Research Center for Pharmaceutical Nanotechnology, Tabriz University of Medical Sciences, Tabriz, Iran.

**Keywords:** Ascorbyl palmitate (AP), Apoptosis, Cytotoxicity, Genotoxicity, Flow cytometry, Real-time PCR

## Abstract

***Purpose:*** Ascorbyl palmitate (AP) is a widely used food additive in food industry. In this study, AP was evaluated for potential cyto-genotoxicity on Human Umbilical Vein Endothelial Cells (HUVECs).

***Methods:*** MTT assay and flow cytometry analysis was used for cytotoxicity evaluation, while genotoxicity was carried out using DAPI staining assays and real time PCR.

***Results:*** The growth of HUVECs was decreased upon treatment with AP in dose-and time-dependent manner. Early/late apoptosis percentage in HUVECs treated with this additive was detected using flow cytometry analysis. Also morphology of DAPI stained HUVECs clearly showed chromatin fragmentation. Furthermore, real time PCR results showed that AP induces apoptosis by up-regulation of caspase-3, 9 and down-regulation of Bcl-2 ratio.

***Conclusion:*** The present results indicated that AP has capability to induce apoptosis in HUVECs and its better to make a thorough analysis about its extensive application in food industry.

## Introduction

**Figure 1:**
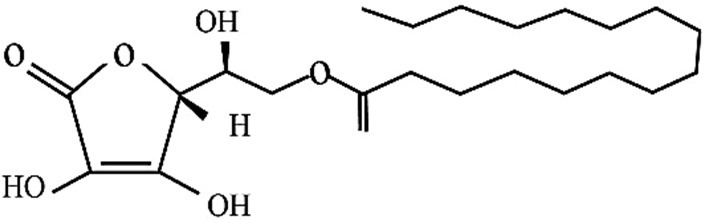
Chemical structure of Ascorbyl Palmitate (AP).

To improve the flavor and taste quality, preservation of appearance during processing, packaging and storage of processed foods, addition of various food additives are required. Although safety and quality of food was protected by food additives but consumers still regard the use of food additives with caution, and even considered food additives as unnatural products with high risks to health.^1-4^ Ascorbyl palmitate (AP) (Figure 1) is an amphiphilic synthetic derivative of ascorbic acid that has widespread use in fat-containing foods, cosmetic, medical and pharmaceutical.^5-7^ It has been approved as food grade antioxidant and generally recognized as safe (GRAS) with the USA Food and Drug Administration (FDA).^8,9^ AP are among the ingredients that listed for use in cosmetic products in the European Union (European Economic Community).^5^ It also was used as color stability agent in meat product. It leads to oxidative rancidity retarding by quenching singlet oxygen.^9^ It was shown that AP in preventing fat and oil oxidation was more efficient than butylated hydroxyanisole and butylated hydroxytoluene.^10^ Also the protective effect of natural antioxidants combination such as tocophrol and AP (50-50) was higher than that of tert-butylhydroquinone.^11^ However, high doses of AP led in toxicity in mice.^12^ It can cause formation of bladder stone and body weight decrease in rats fed with high levels of AP diet.^5^ To the best of our knowledge, Cyto-genotoxic effects of AP on Human Umbilical Vein Endothelial Cells (HUVECs) have not been reported. Therefore, we studied the cytotoxic effects of AP by MTT (3-(4, 5-dimethylthiazol-2yl)-2, 5-diphenyl tetrazolium bromide) and flow cytometry assays. In addition, genotoxicity and DNA damage properties of AP were examined by DAPI (4',6-diamidino-2-phenylindole) staining and real time polymerase chain reaction (PCR), respectively.

## Materials and Methods

### Materials 

HUVECs were attained from national cell bank of Iran (Pasteur institute, Iran). Fetal bovine serum (FBS) and Dulbecco’s modified Eagle’s medium (DMEM) were obtained from Gibco BRL, Life technologies. Annexin V-fluorescein isothiocyanate (FITC) apoptosis detection kit was obtained from Oncogene Research Products (San Diego, CA, USA). SYBR PremixExTaq and Taq DNA polymerase was purchased from Takara BIO (Otsu, Shiga, Japan). RNX-PLUS and primer, DEPC were bought from CinnaGen (Tehran, Iran). PCR buffer and dNTP were obtained from Fermentas (Helsinki, Finland). Ascorbyl palmitate, Trypsin, MTT powders and other chemicals were purchased from Sigma-Aldrich (Steinheim, Germany).

#### Cell Culture

The complete medium containing DMEM, 10% fetal bovine serum (FBS) and antibiotics (100 U/mL penicillin and 10 μg/mL streptomycin) was used for HUVECs culture and then incubated at 37°C in humidified atmosphere containing (95% air and 5% CO_2_). The culture medium was replaced with fresh medium every two days and in confluency of 80–90% cells were passaged. The concentration of AP stock solution was 1×10^3^ M, which was attained by dissolving of proper quantity of AP in filtered media.

#### Cell inhibition assessment

The effect of AP on growth of HUVECs detected using MTT assay. Briefly, the HUVECs were seeded at 15 × 10^3^ per well in DMEM supplemented with 10% FBS and 1% penicillin/streptomycin in 96-well plates. Cells were allowed to attach for about 24 h before being exposed to AP. After 24 h of incubation, they were treated with fresh medium containing 25, 50, 100, 200, and 400 μM of AP and incubated (in 95% air, 5% CO_2_ at 37°C) for 24 and 48 h. Subsequently, 50 μl of MTT dye was added to each well and the plates were incubated at 37°C for 4 h. Afterward medium replaced with the dissolver reagent (100 μL of dimethyl sulfoxide (DMSO)) and the plates were incubated for 20 minutes. Cell viability was evaluated by measuring UV-visible absorbance at 570 nm using a spectrophotometric plate reader.^13^

#### DAPI staining assay

The induction of DNA fragmentation and apoptosis by AP was evaluated by fluorescence microscopic analysis of HUVECs after staining with DAPI. In summary, HUVECs (5×10^5^ cells/well) were seeded in 6-well plates and IC50 concentration of AP (125 μl) was used for treatment of cell. Also DMSO (80 μl) treated HUVECs was used as positive control. After cells washing with phosphate buffered saline (PBS), they fixed with 600 μl/well of 4% paraformaldehyde (PFA) for 30 minutes at 25 °C. Then, HUVECs were permeabilized with 0.1% (w/v) Triton X-100 for 2.5 min and after 3 times washing with PBS, each well were stained with 400 ng/ml of DAPI for 5 minute. DNA fragmentation was evaluated with Cell Imaging Multi-Mode Reader (BioTekCytation™ 5, USA).^14^

#### Annexin V-FITC/PI assay

FITC-labeled Annexin V assay was carried out for detection of early and late apoptosis. Briefly, HUVECs (5× 10^5^) were seeded in 6-well plate and at confluency of 60%-70% treated with IC50 concentration of AP for 48 h. Treated cells washed with PBS buffer (three times) and detached with trypsin and again re-suspended in 100 μl of 1X binding buffer containing 5 μl of Annexin V-FITC after washing with 500 μl 1X binding (three times). Then cells were incubated in the dark at room temperature (25 °C) for 15 min and again were subjected to centrifugation at 1200 rpm for 5 minutes and the supernatant liquid was discarded. Afterward 100 μl of Annexin V binding buffer was added and stained with 5 μl of propidium iodide (PI). The cells were determined using (FACS) Calibur flow-cytometry (Bec-ton Dickinson) by measuring with emission filters in the range of (515-545 nm) for FITC green and (600-660 nm) for PI (red) fluorescence.

#### Real-time PCR Assay

For real-time PCR experiment, HUVECs were seeded in 6-well plate (3× 10^5^cells/well) and after 48 h treatment with AP (IC50 = 125 μl) and with DMSO (40 μl) as positive control, were incubated at 37 °C in‏ 5‏ %CO_2_. The cells were rinsed with PBS, and their total RNA was extracted from each sample using TRIZOL reagent. Through absorbance measurement, RNA concentrations were obtained by UV-vis Spectrophotometer (Eppendorf BioPhotometer) and at 260/280 nm purity of RNA was determined. Complementary DNA (cDNA) was reverse transcribed using cDNA synthesis Kit according to the manufacturer’s manuals. Using specified primers, the caspase3 (forward: 5′-AGAACT GGA CTG TGG CAT TGA G-3′, reverse: 5′-GCT TGT CGG CAT ACT GTTTCA G-3′), caspase 9 (F‏: 5′‏-TGGTGGAAGAGCTGCAGGT-3' R: 5′-TGGGCAAACTAGATATGGCGT-3′‏), bcl-2 (F: 5′- TGG GAT GCC TTT GTG GAA CT-3′, R:‏ 5′‏-TGCCGGTTCAGGTACTCAGT-3′) and GAPDH (F: 5′-CTGGGCTACACTGAGCACC-3′, R: 5′-AAGTGGTCGTTGAGGGCAATG-3′), were amplified. We utilized GAPDH gene as an internal control. After extraction of total RNA and cDNA synthesis, first-step Real-time PCR was carried out on Applied Biosystems real-time thermal cycler according to QuantiTect SYBRGreen RT-PCR kit protocol (Applied Biosystems, UK). For target sequence amplifications, 5 μl of RNA was used per 20μl reaction volume. After performing the real-time PCR run, melting analysis was applied for verification of the amplified product. Per sample, GAPDH (the reference genes) and target genes were amplified using the same run. A serial dilution of total RNA using standard curves illustrated. Real-time thermal condition included holding step at 95°C for 5 min, annealing step at 62 °C for 15 S, 58 °C for 30 S, 54 °C for 30 S and 72 °C for‏ 20 S, and it was continued by a melting step at 95 °C for 15 S, 60 ‏°C for 1 min, and 95 °C for 15 S. The real-time RT-PCR tests were repeated three times.^15-17^

## Results

### Cell inhibition assessment

**Figure 2:**
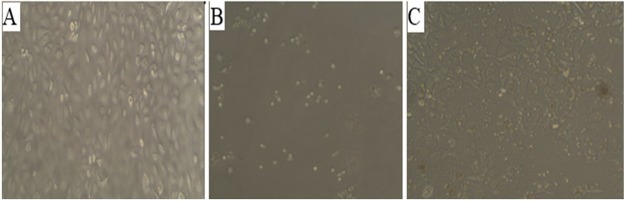
Light microscopy images of HUVECs. (A) Untreated cells (B) Positive control treated with 5% DMSO and (C) AP-treated cells (125 μM at 48h).

**Figure 3:**
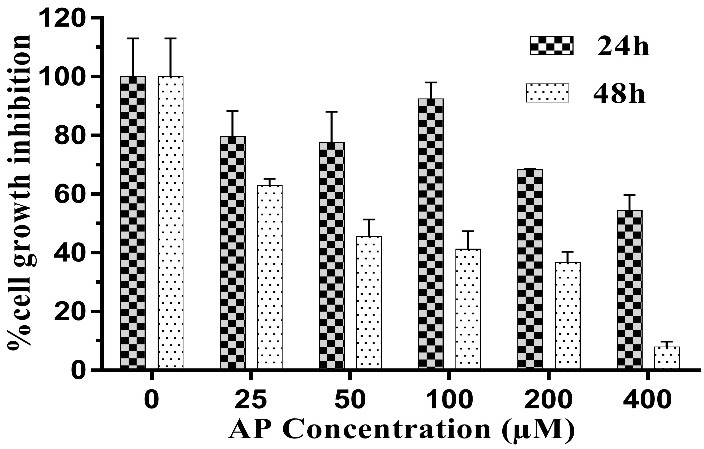
Dose and time-dependent cell growth inhibition in AP treated HUVECs after 24, 48 h.

Microscopic images of treated HUVECs with AP (Figure 2) showed considerable cytotoxicity of AP, which was approved by MTT assay. The potential cytotoxic effect of AP on HUVECs has been shown in Figure 3. IC50 value for AP treated HUVECs after 24 and 48 h incubation were 440 and 125 µM, respectively. The results showed that AP has capability for induction of cytotoxicity in HUVECs in a dose- and time-dependent manner‏.

### DAPI staining

**Figure 4:**
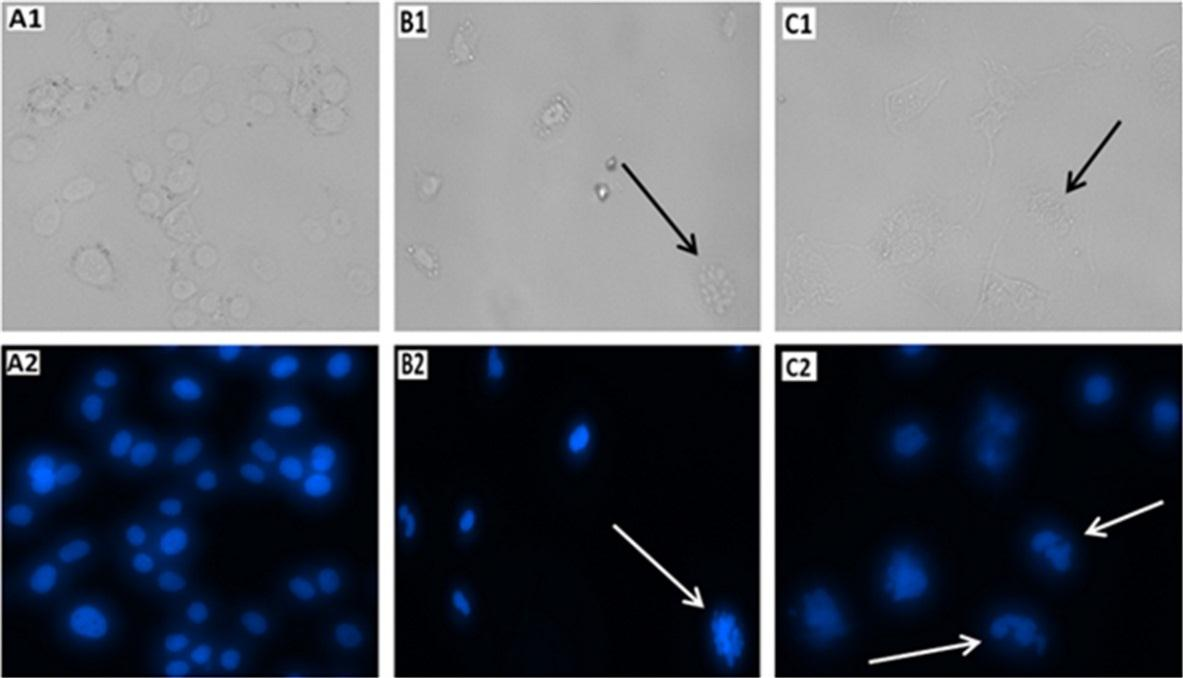
Light and fluorescence microscopy images of HUVECs stained with DAPI (A1 & A2) Untreated (B1 & B2) Positive control treated by 5% DMSO and (C1 & C2) Ascorbyl Palmitate treated cells (125 μM). The arrows demonstrated chromatin and DNA fragmentation occurrence in positive control cells (treated by 5% DMSO) and AP treated cells.

Induction of apoptosis in HUVECs upon treatment with IC50 concentration of AP (125 μM) was evaluated using microscopic analysis of DAPI stained cells. As it is demonstrated in Figure 4, DNA fragmentation which is sign of apoptosis induction is obvious in AP treated cells. Based on the attained results it can be deduced that AP may inhibit proliferation of HUVECs by inducing apoptosis. AP caused a significant effect on nuclear shrinkage and DNA fragmentation in the nucleus of treated HUVECs while the nucleus morphology of untreated cells did not changed.

### Annexin V-FITC/PI assay

**Figure 5:**
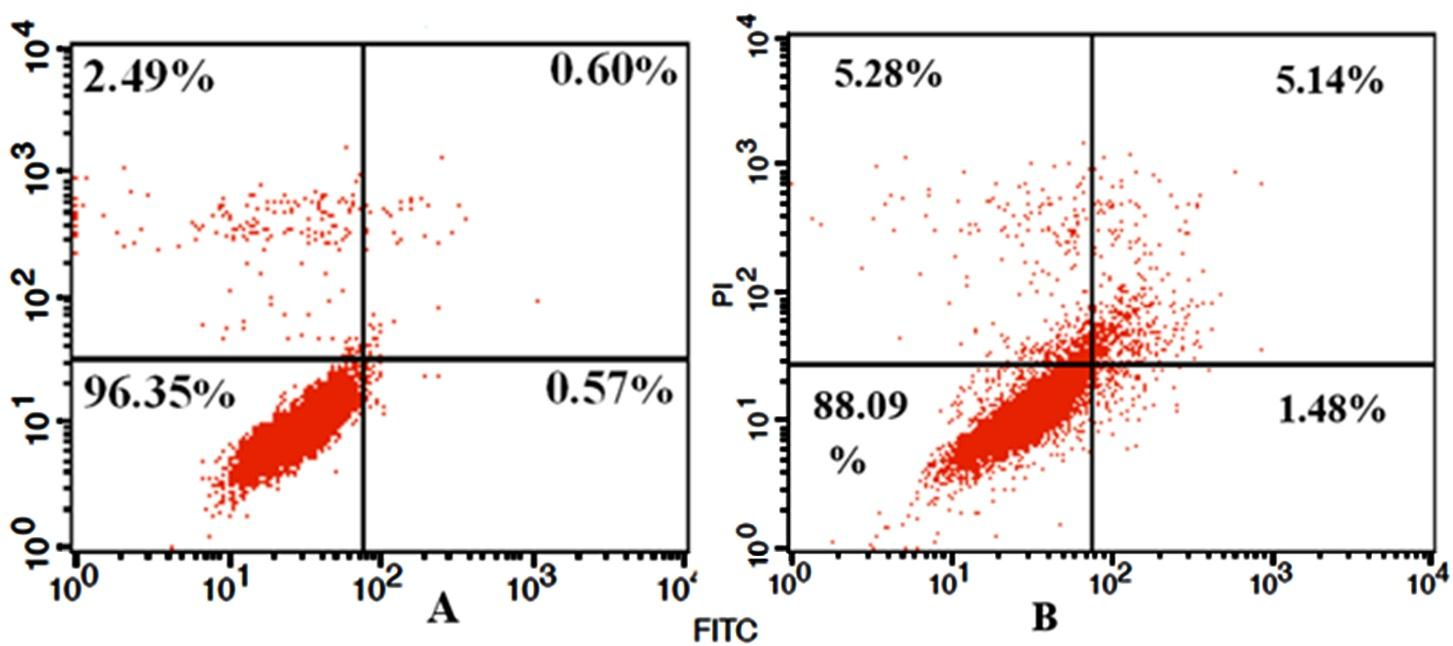
Annexin V-FITC and PI apoptosis detection assay in HUVECs; (A) Untreated control cells. (B) Treated cells with 125 μM of AP. Results show that 5.14% of treated cells with AP were in late stage of apoptosis, 1.48% in early stage of apoptosis and 5.28% of treated cells were necrotic cells.

Cell apoptosis was analyzed using the Annexin V-FITC and PI assay. Figure 5 shows that 1.48% of treated cells with AP were in early stage of apoptosis, 5.14% in late stage of apoptosis and 5.28% of treated cells were necrotic cells. However, only 0.57% and 0.6% of untreated cells were in early and late stages of apoptosis, respectively and 2.49% was necrotic cells for the same period of treatment. Flow cytometry analysis confirmed that the AP can induce cell death through apoptosis.

### BCL_2_ and caspase 3, 9 expression level alterations

**Figure 6:**
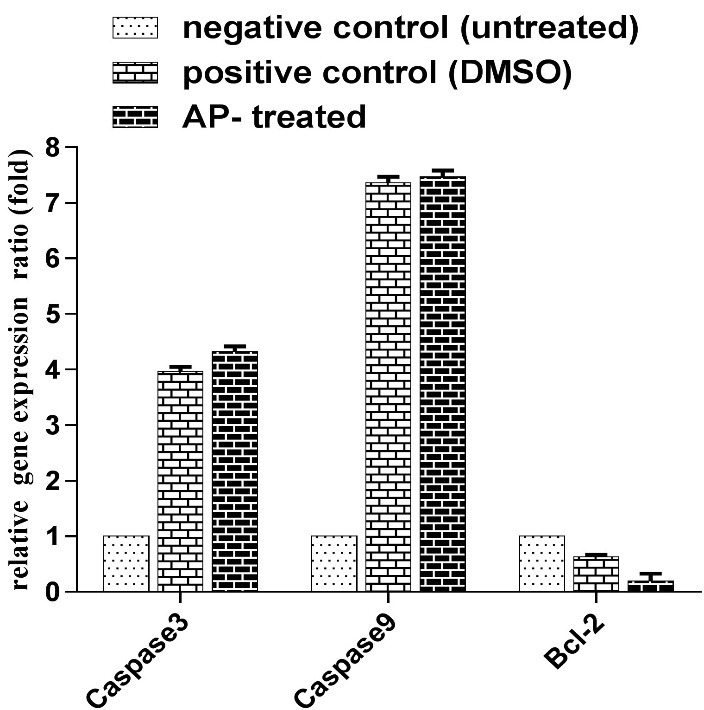
Bar graphs represent the relative expression ratio of Bcl-2 and caspase 3 and 9 calculated for each group (negative control: untreated cell, positive control: DMSO-treated, AP-treated).

The expression levels of the Bcl-2 and caspase 3, 9 mRNA were measured with RT-qPCR (Figure 6). Figure 6 clearly demonstrated that AP induced apoptosis by decreasing Bcl-2 gene expression ratio and increasing caspase-3, 9 activities.

## Discussion

Synthetic food additives such as AP are commonly used as antioxidant in the food industry. Despite the worldwide use of this antioxidant in the food products, its effects and mechanism of action on various cells have not been obviously understood. Thus, in this study we assessed the cytotoxicity and genotoxicity effect of AP additive on HUVECs.

The cytotoxic effect of AP on HUVECs was studied using MTT assay. It was shown that this additive has capability for induction of cytotoxicity in HUVECs in a dose- and time-dependent manner‏. Translocation of the phosphatidylserine (PS) from the inner side to the outer side of the cell membrane upon occurring apoptosis can be confirmed by apoptosis kit. Staining of the cells with a fluorescent conjugate (FITC) of Annexin V that has a high affinity to PS in the cell surface can lead in apoptosis detection. Therefore, the cell death can be detected via flow cytometry technique. In addition, the apoptotic or necrotic cells can be distinguished by both Annexin V-FITC and PI staining of living cells.^16,18^ Briefly, the viable cells are considered as both Annexin V and PI negative ones, while cells that are in early stage of apoptosis are PI negative and Annexin V positive, and both Annexin V and PI positive cells are in late stage of apoptosis. Finally, necrotic cells are PI positive and Annexin V negative ones.^13^ Based on this theory, using FITC-labeled Annexin V flow cytometry, we clearly observed that this additive was able to induce both apoptosis and necrosis in treated cells with IC50 concentration.

Genotoxicity assessment using the DAPI staining assay showed DNA double strand breaks in HUVECs treated with AP. We observed DNA damage and nuclear chromatin fragmentation in HUVECs treated with AP.

Analysis of gene expression in apoptosis plays a major role in many forms of apoptotic cell death. Caspases can be activated through three pathways. The extrinsic (or death receptor) and intrinsic (or mitochondrial) pathways of apoptosis are two frequently explained initiation pathways. Both pathways ultimately results in occurrence of apoptosis. The intrinsic endoplasmic reticulum pathway is known as third initiation pathway.^19^ Up-regulation of caspase 3, 9 and down-regulation of anti-apoptotic survival gene such as Bcl-2 leads in execution of apoptosis.^17,19-21^ Down-regulation of Bcl-2 and up-regulation of caspase 3, 9 confirmed that this additive is able to induce apoptosis within treated cells.

## Conclusion

The cells treatment with AP can stimulate apoptosis and alter gene expression level. The cell inhibition assessment (MTT assay) results revealed that AP was able to induce cytotoxicity in HUVECs in a dose- and time-dependent manner. Significant effect of AP on nuclear shrinkage and DNA fragmentation in the nucleus of treated HUVECs was demonstrated. The happening of early and late stages of apoptosis and even necrosis within AP treated normal cells was verified through flow cytometry analysis. In addition, AP inhibited the growth of HUVECs by inducing apoptosis via up-regulation of caspase-3, 9 and down-regulation of Bcl-2. Finally, based on the achieved results, it can be concluded that this additive at high concentration can be considered as cyto-genotoxic agents.

## Acknowledgments

The authors gratefully acknowledge the financial support of this study by the Tabriz University of Medical Sciences, which was a part of M.Sc thesis No: T/A/124, Tabriz, Iran.

## Ethical Issues

Not applicable

## Conflict of Interest

The authors declare that they have no conflict of interests
